# Bacteria of the Mouth

**Published:** 1899-10

**Authors:** 


BACTERIA OF THE MOUTH.—In the September, 1899, number of the *Dental Review* appears an article from the pen of Dr. Leak Mills, Omaha, Nebraska, under the head of "The Use of Antiseptics and Disinfectants in Dentistry." On page 704 he says: "Along with the study of bacteria as a natural sequence of the same, came the use of antiseptics and disinfectants. Prevention is the watchword in combating the invisible but mighty foes."

Dr. John H. Girdner, in an article on "Disease Germs" in the March *Munsey*, says the old adage should be made to read "an ounce of prevention is worth ten pounds of cure." [Why not make it 16 to 1?—ED.]

I think we can testify that it appears an easier matter to prevent their entrance into soft tissue than to destroy them after they enter. In hard tissue this does not prove wholly true. And yet the papers appearing on the "Prevention of Decay" are sufficient evidence that even here many believe the ravages of bacteria are more readily prevented than repaired.

Bacteria find access to the human organism through the oral and nasal cavities more readily than in any other way. The former by reason of conditions of heat, moisture, and suitable media of development makes it a most favorable location for these accidental bacteria to lodge and develop. That this is true is easily proven by an examination, under the microscope, of saliva from a reasonably well-cared for mouth. When such an examination is made of a mouth receiving little or no care, the number and variety of organisms is little less than startling. Not only the bacteria instrumental in producing decay and disease of the gums, but those producing disease in various parts of the system, the pneumococcus of Frænkel, the Klebs-Loeffler bacillus of diphtheria, the typho bacillus, in fact, most pathogenic organisms, as well as many non-pathogenic, have been found in the oral cavity.

If the gums, or any tissue of the oral cavity are diseased, or the natural power of resistance of any portion of the system be weakened, these accidental germs may thereby be afforded entrance, with serious results. Likewise the swallowing of pus from chronic abscesses, and the toxines produced by bacteria in various diseases of the soft tissue, may produce anæmia, indigestion, and even more serious disorders. Surely then, the responsibility devolves upon us as dentists, to use every means at our command to control these conditions and to impress upon our patients the necessity of proper prophylactic measures on their part. This can not be too strongly urged.

